# Activity quantification and dosimetry in radiopharmaceutical therapy with reference to ^177^Lutetium

**DOI:** 10.3389/fnume.2024.1355912

**Published:** 2024-03-28

**Authors:** Keamogetswe Ramonaheng, Milani Qebetu, Honest Ndlovu, Cecile Swanepoel, Liani Smith, Sipho Mdanda, Amanda Mdlophane, Mike Sathekge

**Affiliations:** ^1^ Department of Medical Physics and Radiobiology, Nuclear Medicine Research, Infrastructure (NuMeRI) NPC, Pretoria, South Africa; ^2^ Department of Nuclear Medicine, Steve Biko Academic Hospital, Pretoria, South Africa; ^3^ Department of Nuclear Medicine, Faculty of Health Sciences, University of Pretoria, Pretoria, South Africa

**Keywords:** ^177^Lutetium, SPECT, PET, theranostics, radiopharmaceutical therapy, patient-specific dosimetry, activity quantification, absorbed dose

## Abstract

Radiopharmaceutical therapy has been widely adopted owing primarily to the development of novel radiopharmaceuticals. To fully utilize the potential of these RPTs in the era of precision medicine, therapy must be optimized to the patient's tumor characteristics. The vastly disparate dosimetry methodologies need to be harmonized as the first step towards this. Multiple factors play a crucial role in the shift from empirical activity administration to patient-specific dosimetry-based administrations from RPT. Factors such as variable responses seen in patients with presumably similar clinical characteristics underscore the need to standardize and validate dosimetry calculations. These efforts combined with ongoing initiatives to streamline the dosimetry process facilitate the implementation of radiomolecular precision oncology. However, various challenges hinder the widespread adoption of personalized dosimetry-based activity administration, particularly when compared to the more convenient and resource-efficient approach of empiric activity administration. This review outlines the fundamental principles, procedures, and methodologies related to image activity quantification and dosimetry with a specific focus on ^177^Lutetium-based radiopharmaceuticals.

## 1 Introduction

The medical community including nuclear medicine (NM) is strongly in favor of personalized treatment. Within the NM field, the recent advancements in molecular medicine have led to a surge in the development of radiopharmaceutical therapy (RPT). Both diagnostic and therapeutic radiopharmaceuticals necessitate dosimetry estimations before clinical use to assess radiation toxicity and overall effectiveness for disease diagnosis or treatment. Dosimetry plays a crucial role in diagnostic nuclear medicine, primarily to evaluate the cancer risk associated with imaging procedures. It entails determining the mean absorbed dose by organs based on representative anatomical models for assessing risk data related to exposed populations (1). In therapeutic applications, dosimetry focuses on organ toxicity and tumor control for individual patients. Ultimately, the medical decision to treat a patient depends on the evaluation of tumors and organs at risk (OAR) (2). Dosimetry data may contribute to adjusting the administered activity for treatment purposes. Based on the dosimetry findings, the administered activity may be increased or decreased for personalized medicine tailored to each patient's needs while minimizing the radiation-induced toxicity to the critical organs and adhering to their threshold radiation doses. The European Council (EC) Directive 2013/59/Euratom establishes fundamental safety standards for protection against the risks associated with exposure to ionising radiation (3). The new EC Directive 2013/59 mandates the justification of medical exposure to ionizing radiation and reaffirms the practice of optimization which may be achieved in close collaboration with medical physicists. According to EC Directive 2013/59/Euratom, radiation exposure for therapeutic purposes should be individually planned and verified to minimize doses to non-target volumes and tissues. The directive also specifies that patients undergoing treatment or diagnosis with radiopharmaceuticals should receive information on the risks of ionizing radiation and appropriate instructions to limit doses to persons in contact with the patient as much as possible.

**Figure 1:**
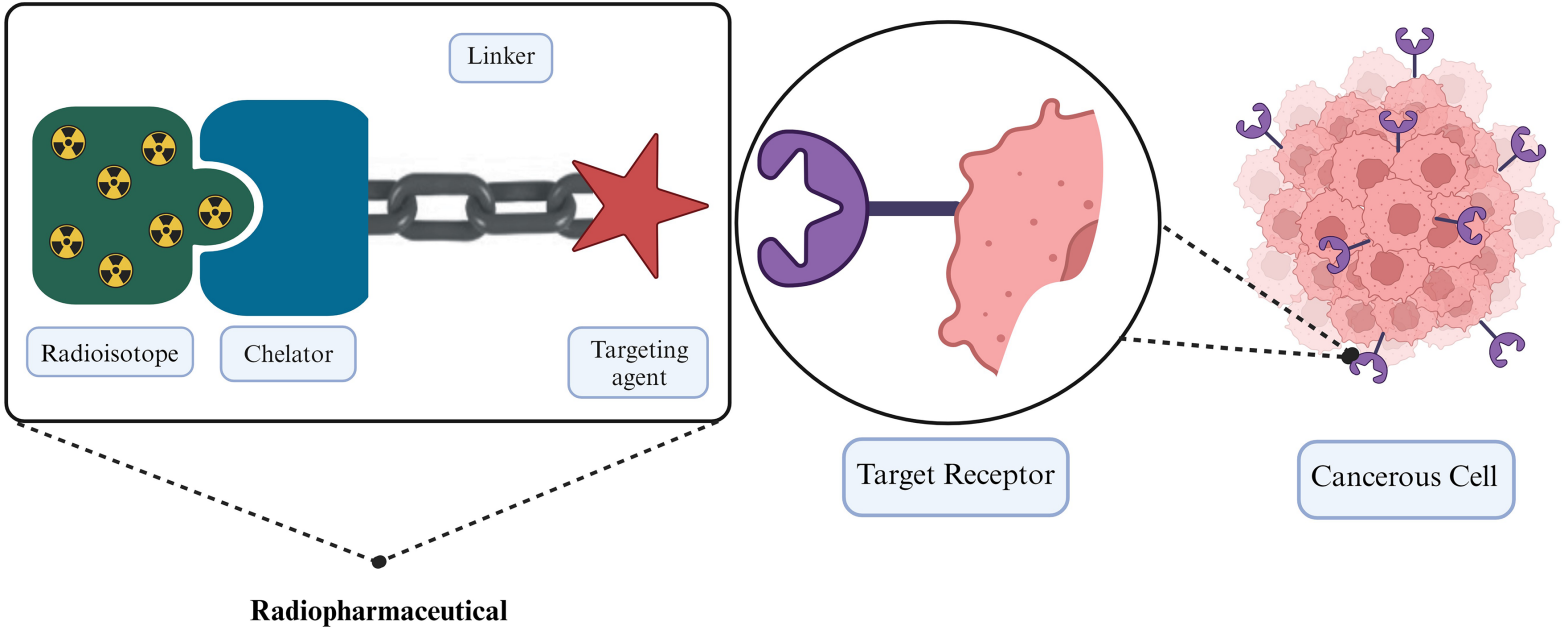
Illustration of the fundamental components in radiopharmaceutical therapy employing a linker to connect a target agent to the radioactive molecule (11).

Patient-specific dosimetry represents a significant opportunity to improve therapeutic outcomes by revealing critical insights into the trade-off between treatment efficacy and patient-specific toxicity. However, to date, patient-specific dosimetry has not undergone a thorough investigation to the full extent of its potential in the optimization of RPT. In this era of personalized therapy, there is a potential to combine RPT and external beam radiotherapy (EBRT) to treat cancers (4). The potential of using combinational therapy has been demonstrated in cancers such as prostate cancer and hepatocellular carcinoma (4–7). Despite both EBRT and RPT employing ionizing radiation for cancer treatment, they exhibit distinct differences, which makes it necessary to distinguish systematic radiation from RPT and EBRT. RPT represents a targeted therapy, where a radionuclide is precisely directed toward its intended target using a pharmaceutical agent specifically designed to bind to the target tissue. The three essential components of RPT involve a radioisotope with the associated chelater, a targeting agent, and a linker that connects the two (8), as illustrated in Figure 1. These RPTs are typically introduced into the bloodstream via intravenous infusion. The targeting agent actively seeks out the intended target i.e., the cancerous cells, and facilitates radiological damage from the energy released by the radionuclide to the cell deoxyribonucleic acid (DNA). This radiation-induced DNA damage may be irreversible, leading to cell death. Cancer cells are notably sensitive to such damage. The cytotoxic effect of the RPT and its extent on surrounding cells is dependent on the physical properties of the radionuclide (9, 10). In the case of beta and alpha particles, the extent to which the emitted radiation can penetrate cells bound to the radiopharmaceutical and surrounding cells varies depending on the specific radionuclide employed and the energy it releases. The radiopharmaceutical must remain within the cell long enough to induce radiation damage.

The phenomenon of RPT is distinguished by the biochemical and physical processes occurring during radiological decay. Crucial patient-specific bio-kinetic data, such as cellular uptake and release, non-uniform distribution within the target, and metabolic behavior, play a pivotal role in determining patient-specific dosimetry (12). RPT employs a continuous, gradually decreasing radiation dose rate. This approach results in a more intricate temporal and spatial distribution of radiation dose when compared to EBRT (12, 13).

**Figure 2:**
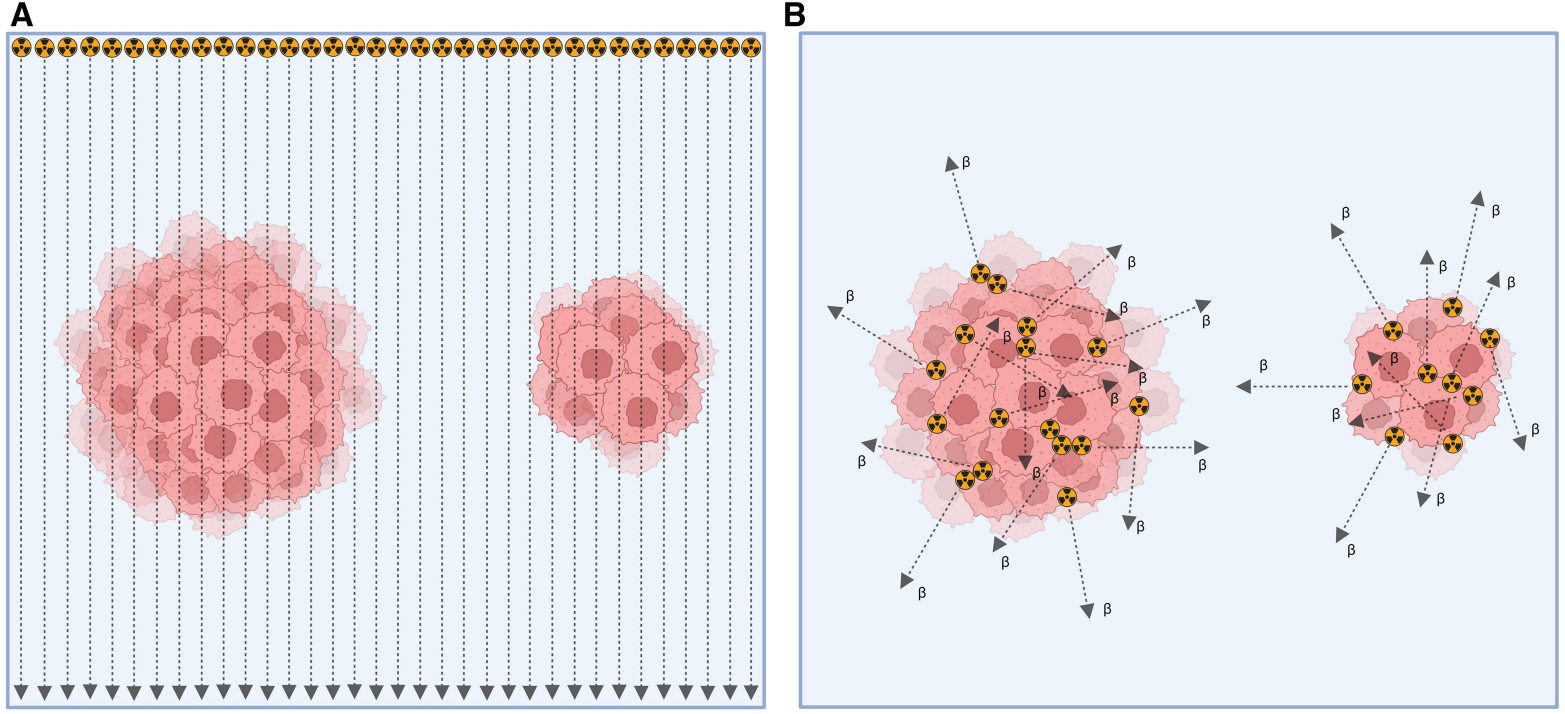
Depiction of tumor cell irradiation with (**A**) external beam radiation therapy and (**B**) radiopharmaceutical therapy (14).

In contrast, EBRT uses fractionated high dose rates to induce lethal damage to cancer cells, combined with non-treatment intervals for cellular repair (12). Another key difference between these modalities, as depicted in Figure 2, is that EBRT administers a consistent absorbed dose per cell, regardless of the number of cells exposed to irradiation. Depending on the size of the tumor, the absorbed dose per cell might be changed at the position where the Bragg-peak occurs. On the other hand, in RPT, the absorbed dose per cell due to internal emissions from neighboring cells depends on the emission ranges of particles and the quantity of targeted cells (14).

The primary objective of RPT is to selectively deliver the highest possible absorbed dose to tumors while minimizing potential toxicity to OAR. Achieving this goal ensures that the absorbed doses in OAR remain below respective tolerance levels to minimize adverse side effects (15). The effectiveness of this treatment approach depends on the targeted accumulation and prolonged retention of the radiopharmaceutical in the tumor cells, thus sparing normal tissue. The degree of cell destruction, in the cancerous and normal cells, is described by the absorbed radiation dose, representing the amount of energy transferred to the target tissue per unit mass. Dosimetry defines this energy deposition within the patient's body. The absorbed dose in a specific tissue, measured in Gray's (joules per kilogram), is a key determinant of the biological effect, such as radiation-induced cell death (16). Consequently, estimating the absorbed radiation dose i.e., dosimetry, is an essential tool for the efficacy of radiation-based treatments.

In EBRT, dosimetry is a well-established technique for routine treatment planning. Parameters in EBRT that are reasonably managed include the treatment site, the target volume to be irradiated, as well as the duration and intensity of the radiation. In contrast, the intricate pharmacokinetics and physical interactions of radiopharmaceuticals on a whole-body (WB) level are part of the internal dosimetry process needed for RPT treatments. These fundamental differences in RPT dosimetry as opposed to EBRT increase the challenge of translating research measurements into standard treatments, which usually depend on the total delivered radioactivity rather than radiation doses absorbed at individual sites (17). In contrast to the focal EBRT, radionuclide therapies are administered systemically and, as a result, can target more widespread diseases (18). Consequently, dosimetry should be given equal importance, as is the case with EBRT, as a driving force for the advancement of radionuclide therapies.

RPT is currently undergoing significant development, driven by the introduction of novel imaging and therapeutic radiopharmaceuticals. Dosimetry plays a critical role in achieving a balance between effectively delivering the maximum dose to tumor cells and minimizing damage to healthy tissues. The unique strength of RPT lies in its ability to image and quantitatively assess the likely biological outcomes of treatment through dosimetry and treatment planning. Clinical trials in their early stages should encompass both imaging and dosimetry to utilise the value of these distinctive RPT features. Through this approach, RPT clinical trials can undergo a comprehensive evaluation and be compared to established therapeutic methods (14). Accurate dosimetry will not only benefit the outcomes of individual patients but also reduce the uncertainties in clinical trials and practice (19). Implementing patient-specific treatment guided by dosimetry in RPT can enhance patient outcomes and survival (20). This approach offers multiple benefits, which include establishing optimal absorbed dose tolerance levels, understanding the dose-response relationship between tumors and normal tissue, as well as comparing dose-response patterns between different radiopharmaceuticals and patients. Personalized treatment is standard in EBRT for improved tumor control and reduced normal tissue toxicity, and this principle should be extended to RPT (12). This aligns with the EC Directive 2013/59/Euratom, which recommends implementing individual dose assessment in RPT (3). Patient-specific dosimetry in RPT has the potential to prevent under- or over-dosing associated with fixed administered activity regimes.

### 1.1 Therapeutic radionuclides with emphasis on ^177^Lutetium

An understanding of the RPT phenomenon requires awareness of three distinct types of radiation: photons (penetrating radiation), electrons, and alpha particles (non-penetrating radiation). Photons are primarily used to image the bio-distribution of RPT but are not employed for delivering localized cytotoxic therapeutic radiation. Typically, therapeutic radionuclides emit short-range charged particles such as beta, alpha particles, and Auger electrons, and possess longer physical half-lives compared to diagnostic radionuclides. Toxicity continues to be a concern with alpha-emitting radionuclides due to the nuclear recoil effect, which results in the release of radioactive daughter nuclei from initial radiopharmaceutical preparations, potentially leading to unintended irradiation of healthy tissues (21). Recoil energy experienced by daughters during alpha decay can be over 100 times larger than the binding energy of any chemical compound. As a result, bond rupture always follows alpha decay, suggesting that released daughters, often themselves alpha emitters, could be significantly harmful. Three strategies are suggested in the literature to mitigate recoil issues. These include encapsulation in a nano-carrier, rapid uptake of the alpha emitters in tumor cells, and local administration through intratumoral injection (22). Managing alpha particles presents challenges primarily linked with radioisotope daughters (23).

Alpha particles and Auger electrons, characterized by higher linear energy transfers (LET), deposit their energy over shorter distances, leading to increased cell death and limited repair of DNA damage (24). Smaller tumors respond well to treatments with Auger electrons and alpha particles (25, 26). The efficiency of beta particles for treating small tumors depends on their energy and penetration depth into the tissue. The range of emitted beta particles should correspond to the size of the tumor, ensuring that minimal radiation dose is delivered to the adjacent healthy tissue (10). Various radionuclides emit both short-range, non-penetrating radiation and longer-range, penetrating radiation, serving the dual purpose of therapy and subsequent imaging to support patient-specific dosimetry. A more extended physical half-life is desirable for prolonged retention, facilitating the cumulative irradiation of the tumor and maintaining a high ratio of penetrating to non-penetrating radiation. The physical half-life of the radionuclide needs to align with the biological half-life of the radiopharmaceutical to ensure effective treatment (27).

Numerous radionuclides that possess the above-mentioned therapeutic decay characteristics are shown in Table 1 and have been documented extensively (24). ^131^Iodine continues to play a significant role as a therapeutic radionuclide, particularly in thyroid gland ablation using [^131^I]-NaI (28). The advancements in RPT, primarily with the introduction of radiolabeled antibodies and molecules like somatostatin analogs and ligands, have resulted in the widespread use of [^177^Lu]Lutetium-PSMA for treating castrate-resistant prostate cancer (CRPC) (29), as well as [^223^Ra]Radium-Chloride (30). Additionally, ^90^Yttrium microspheres have found application in the treatment of both primary and metastatic liver cancer. Given the high biological effectiveness of alpha emitters, there is a growing interest in RPT utilizing radionuclides such as ^227^Thorium, ^212^Lead/^212^Bismut, ^211^Astatine, ^213^Bismuth, and ^225^Actinium (31). Efforts to enhance the specific activity of radionuclides, such as ^153^Samarium, have been explored for potential applications in RPT for metastatic bone palliation (32, 33). There have only been two clinical trials thus far that summarize the clinical circumstances surrounding the less commonly utilized ^211^Astatine with antibodies as one of the carriers (34). These investigations involve, amongst others, colon cancer, glioma leukemic, and neuroblastoma.

**Table 1:** Therapeutic radionuclides and their properties.

Radionuclide	Therapeutic emission	Emission range in tissue (mm)	Radionuclide half-life	Production method
Yttrium-90	β^−1^	5.30	64.1 h	Nuclear Reactor or Y-90 generator
Iodine-131	β^−1^	0.80	8.0 h	Nuclear Reactor
Samarium-153	β^−1^	0.40	46.5 h	Nuclear Reactor
Lutetium-177	β^−1^	0.62	6.6 days	Nuclear Reactor
Astatine-211	*Α*	0.05	7.2 h	Nuclear Rector
Rhenium-186	β^−1^	4.5	3.8 days	Nuclear Rector
Rhenium-188	β^−1^	11	16.9 h	Nuclear Reactor
Strontium-89	β^−1^	8	50.5 days	Nuclear Reactor
Terbium-161	β^−1^	3	6.95 days	Nuclear Reactor
Tin-117m	Conversion electrons	0.3	14 days	Nuclear Reactor
Lead-212	β^−1^/α	<0.10	10.6 h	Generator
Bismuth-212	β^−1^/*α*	<0.05	1.0 h	Generator
Radium-223	*Α*	0.05–0.08	11.4 h	Generator
Bismuth-213	β^−1^/*α*	2.1	46.6 min	Generator
Thorium-227	*Α*	0.05–0.08	18.7 days	Generator
Copper-67	β^−1^	2.2	2.58 days	Cyclotron
Actinium-225	*Α*	0.05–0.08	10 days	Cyclotron or generator

References (14, 35–39).

[^177^Lu]Lutetium-DOTA-TATE/NOC/TOC has been used in RPT applications to treat late-stage neuroendocrine tumors (NETs) with extensive metastases while [^90^Y]Yttrium-DOTATOC is less frequently employed (40–42). This choice is attributed to ^177^Lutetium's favorable decay characteristics and compatibility with peptide labeling, making it an ideal candidate for the treatment of NETs. RPTs using ^177^Lutetium-based radioligands have the advantage that they can be applied to primary and metastatic cancers with relatively low toxicity (14).

^177^Lutetium has been proven as an established RPT radionuclide due to its application in treating patients with NETs and CRPC. Even though several isotopes are accessible for use in radionuclide treatment applications, ^177^Lutetium persists as the preferred radionuclide used routinely in clinics (43–45). It also has the additional benefit of being readily available commercially. As a result, our emphasis on activity quantification and dosimetry will focus on ^177^Lutetium.

#### 1.1.1 ^177^Lutetium decay characteristics

**Figure 3:**
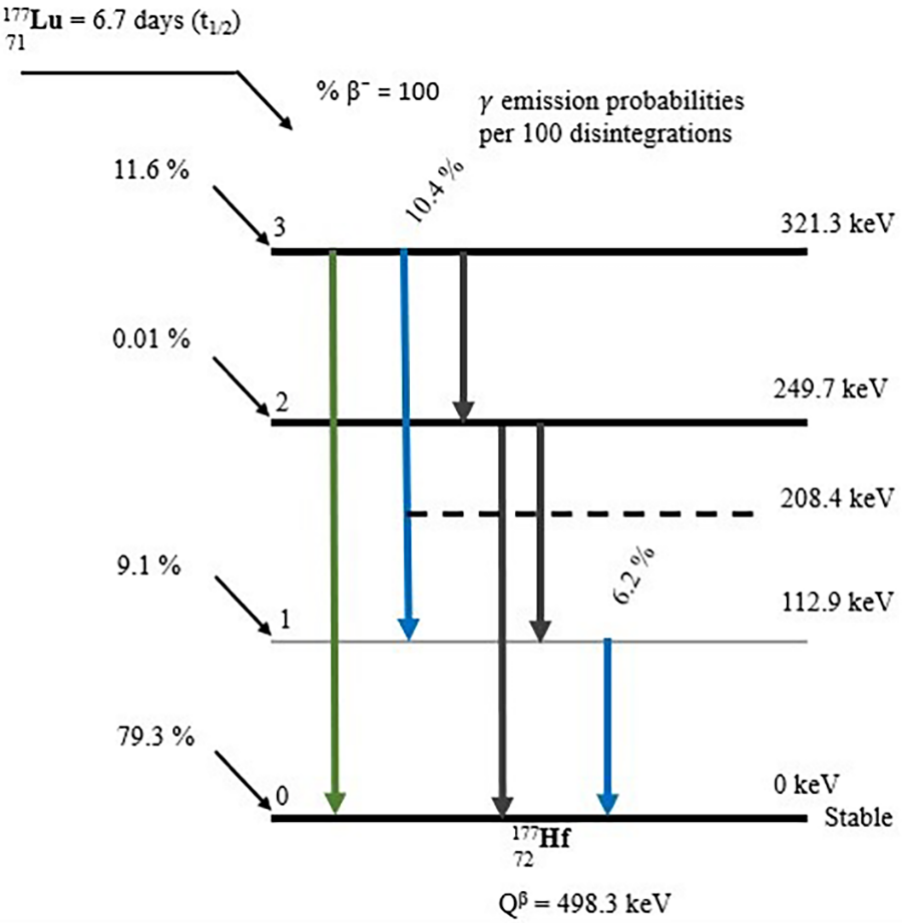
A Beta decay scheme depicting the emission process of ^177^Lutetium (47).

With a physical half-life of 6.7 days and three excited levels above the ground state, ^177^Lutetium decays by beta emission to the stable ground state of ^177^Hafnium (depicted in Figure 3). The physical half-life is sufficiently long to ensure that transportation, storage, and delivery are manageable, yet it is short enough to spare OAR from excessively high doses (46). The highest energy (*E*_max_) that may be produced through Beta emission is 498.3 keV (79.3%) (47). 12.0% of the emissions involve Beta particles with an *E*_max_ of 177.0 keV, which yields an excited state of ^177^Hafnium at 321.3 keV above the ground state. Furthermore, Beta particles with an *E*_max_ of 385.4 keV (9.1%) are encountered, leading to an excited state of ^177^Hafnium at 249.7 keV above the ground state. The decay process produces trace amounts of Auger electrons and x-rays. ^177^Hafnium de-excites to the ground state by emission of gamma rays, with energies most prevalent at 112.9 keV (6.2%) and 208.4 keV (10.4%). At energies of 54.6 keV (1.6%) and 55.8 keV (2.8%), characteristic x-rays are obtained (47). Owing to the Beta particles' interaction with the tissue, the Bremsstrahlung yield is extremely low (>11%), with the majority of the photons (∼85%) having energies below 50 keV (48).

The two above-mentioned gamma rays have been effectively employed for imaging (49) to facilitate dosimetry (50). The Beta particles (*E*_max_ of 498.3 keV) are successfully applied in RPT (51) and have a short mean soft tissue penetration depth of 0.7 mm (52). The Beta particles from ^177^Lutetium decay ensure a uniform absorbed dose at the cellular level due to the high energy while still avoiding harm to the surrounding healthy tissues (53).

### 1.2 Theranostics with ^177^Lutetium

**Figure 4:**
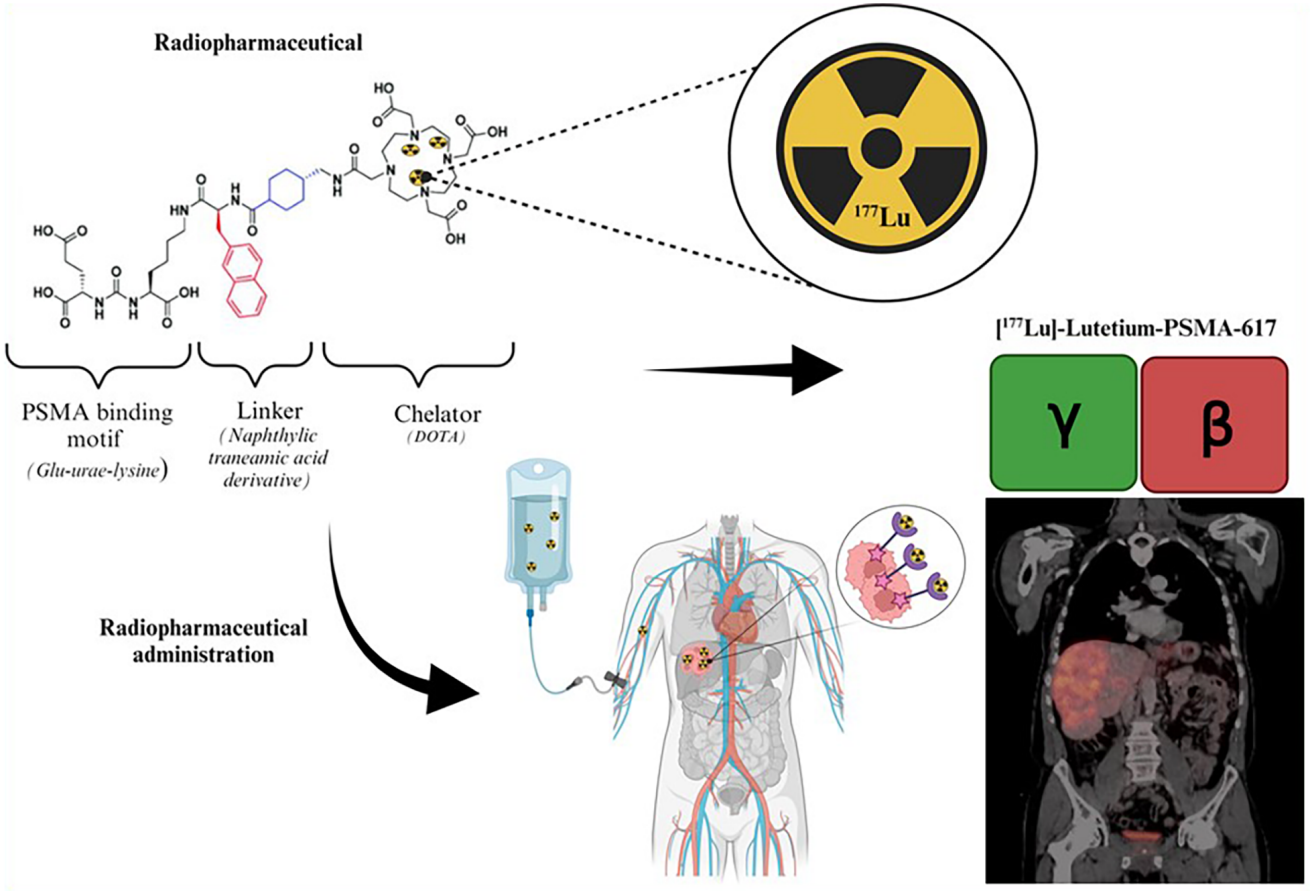
Illustration of the theranostic direct approach using [^177^Lu]Lutetium-PSMA (55).

^177^Lutetium is considered a theranostic radionuclide since it can be used to assess tumor uptake and cancer progression and for treatment purposes (54). The theranostic approach with ^177^Lutetium employed for personalized patient management may be direct or indirect. The direct method takes advantage of ^177^Lutetium's emission of both gamma and beta radiation as demonstrated by Figure 4. This gamma radiation can be employed for image-based treatment planning before sequential treatment cycles and for RPT. This method employs image-based dosimetry from the preceding treatment to predict potential radiation-induced adverse side effects and to ensure that the doses received by the OAR remain within acceptable tolerance levels. This approach determines the feasibility of administering additional dose cycles following the initial ^177^Lutetium therapy. The use of image-based indirect theranostic approaches with [^68^Ga]Gallium-PSMA or [^68^Ga]Gallium-DOTA-TATE/NOC/TOC is employed to diagnose patients with CRPC and NETs, eligible for [^177^Lu]Lutetium-PSMA and [^177^Lu]Lutetium-DOTA-TATE/NOC/TOC treatment respectively.

#### 1.2.1 ^177^Lutetium dose-limiting factors and use in neuroendocrine tumors and prostate cancer

##### 1.2.1.1 [^177^Lu]Lutetium-somatostatin receptors

The use of [^177^Lu]Lutetium-DOTATATE for the treatment of patients with midgut NETs was approved as a result of the NETTER-1 trial. The dose-limiting OAR for RPT of [^177^Lu]Lutetium- DOTATATE in patients with NETs are the kidneys and bone marrow (56). The activity and the number of cycles administered for RPT are governed by the tolerance values of the OAR. The decision to administer a subsequent cycle relies on the estimated absorbed dose determined from the dosimetry data of the preceding cycle, to ensure that the cumulated dose does not exceed the tolerance levels of the OAR (57). Furthermore, randomized clinical trials depend on the threshold tolerance values of the dose-limiting organs applicable to RPT. Since acute hematological toxicity is common, the administration of additional cycles during treatment may need to consider bone marrow deficiency. The permissible maximum absorbed dose to the bone marrow is 2 Gy (58). Although bone marrow involvement is taken into account, it is not the primary organ that limits the dose administered (59). The most critical OAR for [^177^Lu]Lutetium-DOTATATE RPT is the kidneys. This is owing to the proximal tubular reabsorption and retention of the radiopeptide in the interstitium and subsequently renal irradiation. Positively charged amino acid infusion may reduce kidney doses to some extent by lowering high renal retention and limiting proximal re-absorption (60). Since the kidneys are a late-responding tissue and the radiation-induced harm from deterministic effects may not become apparent for one year or more, it is critical to monitor kidney dosage and kidney function in patients. This finding prompted a research cascade in renal dosimetry of [^177^Lu]Lutetium RPT (61–67).

It has been commonly accepted that kidney doses obtained via RPT should not exceed the maximum tolerance threshold of 23 Gy (68). This value is based on the fractionated EBRT tolerance (69) which results in a 5% probability of developing late kidney damage in 5 years. Garkavij et al. (56) contended that the suggestion might be called into doubt due to the notable differences in the absorbed dose rate between EBRT and RPT. For this reason, the application of dose-response data applied to RPT has been reviewed (70–72). The tolerance was raised to 27 Gy in research by Valkema et al. (73), citing the fact that EBRT provides a dose rate that is significantly higher than RPT. Clinical trials have been carried out where additional treatment cycles are administered until the calculated biological effective dose (BED) for the kidneys reaches either 27 Gy or 40 Gy, with the choice between these levels determined by patient-specific risk factors (74). Despite these debates, the generally accepted upper limit of kidney dose that can be administered with RPT remains 23 Gy. Evidence-based trials would need to demonstrate that patient-individualized RPT with [^177^Lu]Lutetium-DOTATATE is superior to the 7.4 GBq over the four-cycle standard as indicated in the NETTER trial (75).

##### 1.2.1.2 [^177^Lu]Lutetium-PSMA-617

The use of [^177^Lu]Lutetium-PSMA-617 for the treatment of patients with metastatic CRPC has been approved by the FDA as PluvictoTM after the results of the VISION trial (76). The dose-limiting organs for [^177^Lu]Lutetium-PSMA-617 are similar to those of [^177^Lu]Lutetium-DOTATATE with a potential concern of xerostomia radiation-induced effects on the salivary glands. Similarly taken from EBRT data, the tolerance threshold of the salivary gland is based on EBRT data as 25 Gy to ensure a less than 25% probability of long-term radiation damage. Salivary gland dysfunction varies amongst patients and dose reduction measures such as hydration can mitigate the radiation-induced effects (77). Radiation-induced salivary gland toxicity has been found to have minor clinical relevance for [^177^Lu]Lutetium-PSMA (78).

The response variation of patients treated with 7.4 GBq of [^177^Lu]Lutetium has been ascribed to the comparatively low activity of “one dose fits all” (79). For this reason, RPT finds itself at a crossroads between patient-specific treatment and the rigid “one dose fits all” fixed activity regime. Table 2 outlines the vectors explored with the [^177^Lu]Lutetium treatment. Among these vectors [^177^Lu]Lutetium-PSMA-617 and [^177^Lu]Lutetium-DOTATATE are extensively used.

**Table 2:** Vectors labeled with [^177^Lu]Lutetium for potential use in radiopharmaceutical therapy.

Lu-177 Targeting vectors		
Peptides: PSMA-617, I & T		
Administered Activity	Target Cancer	Organs at Risk
5.5–7.4 GBq in humans	Castrate-resistant prostate cancer	Bone marrow, salivary glands, and kidneys
Peptides: DOTA-TATE, DOTA-TOC, DOTA-NOC		
Administered Activity	Target Cancer	Organs at Risk
5.5–7.4 GBq in humans	Neuroendocrine tumors and metastasis	Bone marrow and kidneys
Bone-seeking tracers (Bisphosphonates): EDTMP and MDP (Trial)		
Administered Activity	Target Cancer	Organs at Risk
3.8 GBq in humans	Bone metastases	Liver, kidneys and red marrow
Peptidominetics: PP-F11N, NMG1, NMG2 and NMG3 (Trial)		
Administered Activity	Target Cancer	Organs at Risk
20 MBq in mice	Small cell lung cancer and medullary cancer	Stomach and kidneys
Monoclonal Antibodies: J591 (Trial)		
Administered Activity	Target Cancer	Organs at Risk
2.8 GBq in humans	Castrate-resistant prostate cancer	Bone marrow, spleen, liver and kidneys
Monoclonal Antibodies: Rituximab, Tetulomab and Cetuximab (Trial)		
Administered Activity	Target Cancer	Organs at Risk
20 MBq/kg in mice for rituximab and tetulomab	Non-Hodgkin lymphoma and head and neck	Bone marrow, spleen, liver and kidneys
14.8 MBq/kg for cetuximab		
Monoclonal Antibodies: huA33 (Trial)		
Administered Activity	Target Cancer	Organs at Risk
15 MBq	Colorectal Cancers	Liver, spleen, kidneys, and bone marrow

EDTMP, ethylenediamine tetramethylene phosphonic acid; PSMA, prostate-specific membrane antigen; huA33, humanized monoclonal antibody A33; NMG 1–3, novel minigastrins 1–3 (80–92).

#### 1.2.2 [^225^Ac]Actinium-PSMA-617 referencing [^177^Lu]Lutetium-PSMA-617

Notably, in instances where therapy with the beta-emitting [^177^Lu]Lutetium-PSMA-617 has failed, RPT targeting PSMA with the alpha-emitting [^225^Ac]Actinium-PSMA-617 has demonstrated therapeutic success (93). This has the benefit of the higher LET from the alpha particles, that if the radionuclide is coupled to a vector that targets antigens expressed on tumor cells, the radiation from the alpha particles can be efficiently delivered to the malignant cells. In this manner, the malignant cells will receive high energy delivery with minimal radiation harm to the surrounding healthy cells, which has been beneficial in the treatment of prostate cancer (94–96). To achieve reliable dosimetry data, the dose deposition should be accurately described to the range of the dose-depositing particles.

The emission range of the RPT particles varies, from millimeters and micrometers to nanometers for beta, alpha, and Auger electrons respectively. Dosimetry computations may be cumbersome with alpha particles due to their limited tissue range and high LETs, resulting in a high-energy deposition close to the emission site. Since dosimetry is computed from positron emission tomography (PET) and single=photon emission computed tomography (SPECT) images the adaption to RPT with alpha-emitting radionuclides administered with low activities leads to low signal-to-noise ratios from the gamma rays, causing image quality challenges (97, 98). These challenges are also brought on by the decay properties of alpha emitters, such as ^225^Ac, with limited gamma emission and the competing bremsstrahlung radiation (99).

Previous dosimetry on [^225^Ac]Actinium-PSMA-617 and [^225^Ac]Actinium-PSMA-I&T extrapolated the uptake of respective ^177^Lutetium-labeled analogs on imaging to mitigate the imaging challenges with alpha emitters (99–101). The derivation of time activity curves (TAC) from [^177^Lu]Lutetium-PSMA-617 data extrapolated to the physical half-life of [^225^Ac]Actinium-PSMA-617 by assuming instantaneous decay of daughter nuclides results in uncertainty of bio-kinetic data at the cellular level (102). Alternatively, the dosimetry of [^213^Bi]Bismut-PSMA-617 was previously determined by extrapolating the uptake of [^68^Ga]Gallium-PSMA-617 from PET images (103). Recent investigations have demonstrated that the degree of uptake with radiolabeled PSMA-617 varies depending on the radionuclide (104, 105). This suggests that an independent assessment of the uptake of [^225^Ac]Actinium-PSMA-617 would improve its dosimetry.

Because alpha particles have short ranges in tissue, small-scale dosimetry techniques such as microdosimetry and autoradiography have been identified to determine the dose distribution on sub-organ levels (97, 106–110). To date, the integration of small-scale dosimetry into clinical practice has not been achieved (14, 15). This is because the activity distribution needs to be quantified to the cellular level for the range of the alpha particles. Even with voxel-based dosimetry, source-target combinations on this scale are challenging to measure, and the distribution of activity over time needs to be quantified at the subcellular level (1). In clinical practice, the quantitative information used as the input data for dosimetry is conventionally obtained from PET and SPECT scans, which have spatial resolution in the order of millimeters. For these reasons, preclinical validations have benefited more from sub-organ dosimetry (107, 111, 112).

### 1.3 Clinical intent of dosimetry

The approach to RPT should be tailored to match the intended therapeutic aim, which could be curative or palliative (113). For a treatment intended to increase the chances of a cure, the focus would be on delivering a substantial therapeutic dose within a relatively short timeframe. The biological response to RPT is determined by the absorbed dose and the LET (113, 114). Individualized administered activity in RPT has been referred to as a “quest for the holy gray” because there are no threshold values from RPT and dosimetry is based on EBRT threshold values (12). Although RPT dosimetry has undergone tremendous effort, concerns such as “to what extent is dosimetry needed for RPT applications” arise from the threshold uncertainty (115). In this exciting era of innovative RPT agents with the possibility of precision medicine, it is still up to the NM community to optimize and customize patient treatment. Initiating patient-specific dosimetry for personalized administered activity would pave the ground for this endeavor considering the therapeutic objective of RPT, whether it is for palliative or curative purposes.

Image quantification of activity distributions and absorbed dose modeling are two aspects of dosimetry. The basic requirements of dosimetry are to determine the radiopharmaceutical bio-distribution and calculate the absorbed dose to the organs of interest based on this distribution. There are numerous processes involved in the procedures used to obtain the bio-distribution data, and the assumptions made vary greatly depending on the protocols and radiopharmaceuticals. Some of the factors contributing to the relatively low acceptance of dosimetry include the large variety in absorbed dose techniques and the predicted “tolerated holy gray.” A foundation for standardizing dosimetry methods is provided by an understanding of the importance and constraints of each step in the clinical dosimetry chain.

## 2 Quantification of image-based activity for dosimetry

**Figure 5:**
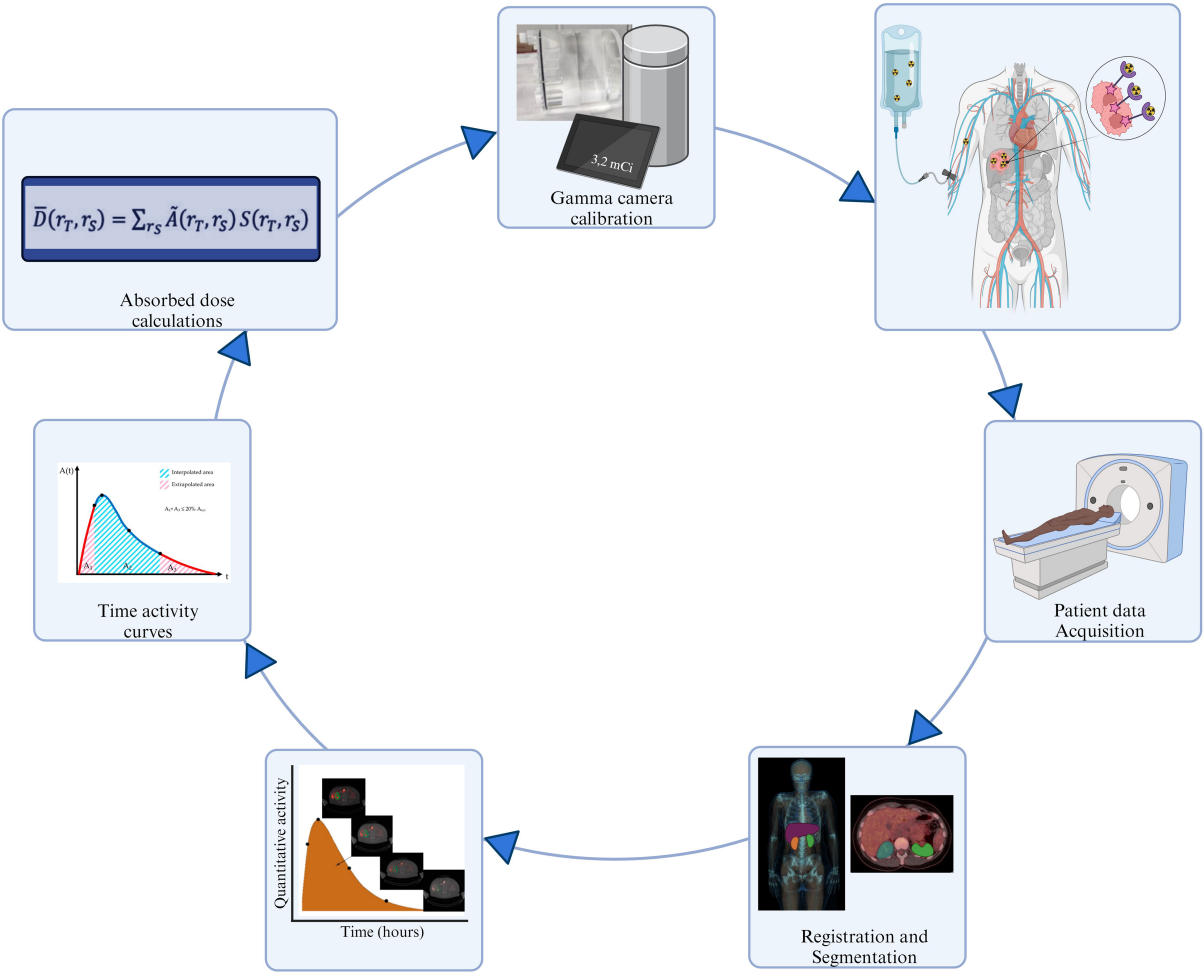
Fundamental steps in the image-based dosimetry workflow.

The quantities for image-based dosimetry include a) the number of decays in the organs of interest for the various times post-administration, b) the decay particles' temporal pattern which determines how the energy is released from the time-activity data, and c) the absorbed energy in the respective mass or volume of the organ of interest. Since the absorbed dose to the radiation-exposed organs is determined from the image-based activity distributions, it pertinently follows that the accuracy of the activity quantification is a crucial factor for dosimetry. A series of steps is required for the calculation of absorbed doses. These steps form part of a chain, illustrated in Figure 5. The process begins with (i) measuring activity in the dose calibrator and obtaining a gamma camera calibration factor, followed by (ii) acquiring patient data through a designated imaging protocol. In the case of SPECT/CT acquisitions, the activity distribution is quantified from the reconstructed corrected registered images which are (iii) segmented using a volume of interest definition to derive absolute activity quantitative data from organs of interest by applying a previously determined calibration factor. Subsequently, (iv) a time-activity curve (TAC) is generated, and TAC analysis and integration are performed to produce a time-integrated activity (TIA). Finally, (v) absorbed dose computations are conducted.

Every step propagates the dosimetry inaccuracy and precision. The foundation for achieving accurate dosimetry lies in the accurate quantification of activity, which is dependent on how faithfully the images represent the true activity distribution. SPECT/CT has overcome the challenges associated with planar imaging. When SPECT/CT modalities are used, the corrections for image degrading factors are carried out as part of the clinical workflow. Therefore, the discussion on image acquisitions shall be made with reference to SPECT/CT data.

### 2.1 Gamma camera calibration factor

SPECT images are generally considered to be non-quantitative, in contrast to PET images. For images to be used for quantification and dosimetry purposes, SPECT images must be obtained in units of activity concentration (kBq/cm^3^) rather than the conventional counts. For certain gamma cameras, to obtain SPECT images in absolute units of activity concentration, a calibration factor (CF) must still be applied to the reconstructed image. The system sensitivity is frequently used as a CF in SPECT images to gain quantitative data (116). It is determined from a planar image of a Petri dish in the air with known activity. The accuracy of the dose calibrator used to quantify the activity for the CF also determines the effective activity administered to the patient, which is another crucial factor to take into account (117). To increase the accuracy of CFs, a variety of geometries have been explored (118).

For activity quantification, the process of obtaining the CF ought to approximate the method used in the quantified clinical studies. The CF source should provide reconstruction and compensation techniques for SPECT applications. This enhances quantitative accuracy and lessens the impact of inaccurate scatter and attenuation corrections (119). The CF measurement is a function of the source geometry and depends on how the volume used to obtain the counts for the CF is defined and incorporated with the recovery coefficient (RC) (120). Techniques for image-based CF that employ SPECT/CT and corrected planar patient data have been presented and compared with conventional phantom CF (121). For the patient data CF, the authors discovered an error rate of less than 2%, resulting in an overall quantification accuracy of 7%.

Establishing a standard for absolute quantitative SPECT by harmonizing the CF in a multicenter and multivendor setting where reconstruction techniques are established is a key step to standardizing dosimetry. Such a multicentre and multivendor study demonstrated that a high patient body mass index (BMI ≥ 47 kg/m^2^) increased CF variability between systems and made it more difficult to quantify minor lesions (less than 10 mm^3^) (122). A standard CF, for gamma camera vendors and models, can be used as input for absolute SPECT quantification in radionuclide therapy research; this will aid with complex clinical dosimetry, especially in multi-center research endeavors. To support this, image voxels from reconstructed SPECT images have recently become available in radioactive concentration units in recent gamma cameras such as the Siemens Symbia IntevoTM scanner (Siemens Healthineers). Concerning PET imaging, the PET systems are usually calibrated to measure accurate concentrations of ^18^Fluorine. The reconstructed standard uptake value (SUV) should be checked for the radionuclide in question (123). For radionuclides other than ^18^Fluorine the reconstruction should incorporate the physical characteristics of the radionuclides to obtain accurate quantitative images.

### 2.2 Image acquisition protocol

**Figure 6:**
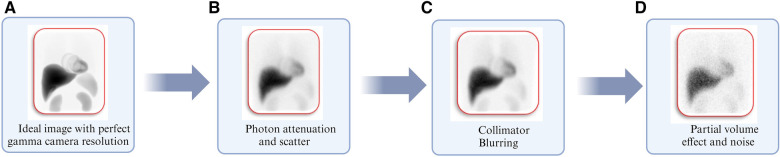
Illustration of degrading physical factors inherent with gamma camera imaging using Monte Carlo simulations corresponding to (**A**) image simulated with perfect gamma camera resolution. (**B**) Image including degradation from photon attenuation and scatter. (**C**) Image obtained in “b” including normal gamma camera resolution. (**D**) The image obtained in “**C**” includes partial volume effects and clinically realistic noise levels (127).

Patient dosimetry is based on gamma camera images that have been tainted by uncertainties in the imaging procedure and the related protocols. Figure 6 depicts various physical factors that degrade the gamma camera images from a perfect representation of the activity distribution within the body, as shown in Figure 6A. These factors include Photon attenuation (absorption) and Compton scattering (scatter), the effects of which are demonstrated in Figure 6B. Figure 6C illustrates the effects of collimator-detector response (CDR) or collimator blurring, while Figure 6D demonstrates partial volume effects (PVEs) and noise. The influence of these variables on patient dosimetry is contingent upon the gamma camera's chosen imaging protocol and the corrections applied to account for these degradation factors. For this reason, image activity quantification has been a subject of investigation for many years (124–132). Over the years, there has been consistent progress in improving the accuracy of activity quantification and image analysis (120, 133–138).

Selecting the appropriate collimators is the first step toward optimizing the imaging protocol. This is based on the energy of the gamma-ray to be imaged and the trade-off between sensitivity and spatial resolution. In the case of ^177^Lutetium, the imaging protocol, the collimator, the imaged gamma-ray energy, and energy window settings have been thoroughly investigated using Monte Carlo (MC) simulations (127). The 208 keV photopeak should be used with a medium energy (ME) collimator and a 20% energy window setting (49, 56, 67, 118, 127, 139–144). Due to the low gamma-ray emission of ^177^Lutetium, the effects of dead time are minimal and imaging can commence immediately after therapeutic activity values are administered (145).

### 2.3 SPECT image activity quantification

Traditionally, 2D planar anterior and posterior WB images have been used for activity image quantification in dosimetry (146–149). Planar WB methods offer a quick and simple method to image the patient's entire body and extract biodistribution information. However, organ overlap, superimposed background activity, and the absence of organ volume information are known drawbacks of planar imaging. When compared to planar images, SPECT images have shown superior image quality and improved quantitative accuracy (150–152). Planar images are often considered to have poorer quantitative accuracy since not all centers consistently undertake additional image acquisitions such as blank and transmission scans. These additional scans are used to compensate for attenuation and further analysis of the images in sub-windows are necessary for scatter corrections. Hybrid planar WB SPECT/CT imaging techniques, also known as hybrid WB/SPECT images, have resolved the trade-off between the faster multiple biokinetic image data collections for dosimetry that planar imaging advocates and the enhanced accuracy provided by SPECT/CT images (153–155). Even though SPECT/CT has overcome the challenges associated with planar imaging, the reconstruction algorithm's integrity and the impact of the inherent image acquisition degradation factors complicate SPECT imaging. Therefore, several steps must be taken to increase the SPECT quantitative accuracy, which is covered in more detail in the following sections.

#### 2.3.1 SPECT image reconstruction

Iterative reconstruction algorithms such as the maximum a posterior (MAP) (156), maximum likelihood expectation maximization (ML-EM) (157), and ordered subset expectation maximization (OS-EM) (158) can include modeling of the physical characteristics of the imaging process. These algorithms mainly consist of compensations for collimator and object scatter, system geometry, and finite detector resolution. These algorithms result in reconstructed images with better image quality and quantitative accuracy and are less prone to artifacts compared to analytical methods such as filtered back projection (159). The OS-EM algorithm has become a standard algorithm with most clinical SPECT processing units. It is highly recommended and commonly used to obtain improved quantitative SPECT data (119, 160). When using the OS-EM reconstruction algorithm, it is important to consider the optimum number of updates, defined as the product of the number of subsets and iterations for a particular SPECT system's reconstruction algorithm. The trade-off is that more updates lead to higher levels of image noise but yield more accurate quantitative activity distributions from the reconstructed images (127, 161, 162). To improve image quality and activity quantification accuracy, the current 3D OS-EM reconstruction algorithms available with most SPECT systems include, in addition to attenuation and scatter corrections, compensation for CDR.

Reconstructing an image from raw projection data is an inverse problem. Although artificial intelligence (AI) technology, particularly deep learning-based solutions, has emerged as a promising solution to the reconstruction of emission images, the inverse problem remains unsolvable with AI. Three distinct systems, namely static scan, dynamic scan, and hybrid fusion, have realized the introduction of AI applications centered around NM image reconstruction (163). AI essentially provides a mapping connection to address specific significant reconstructive challenges, such as completing the transition from the sinogram domain to the image domain or substituting for regularization in traditional algorithms with a data-driven approach. The majority of the image reconstruction work in NM using AI technology is in PET reconstruction (164).

#### 2.3.2 Attenuation correction

**Figure 7:**
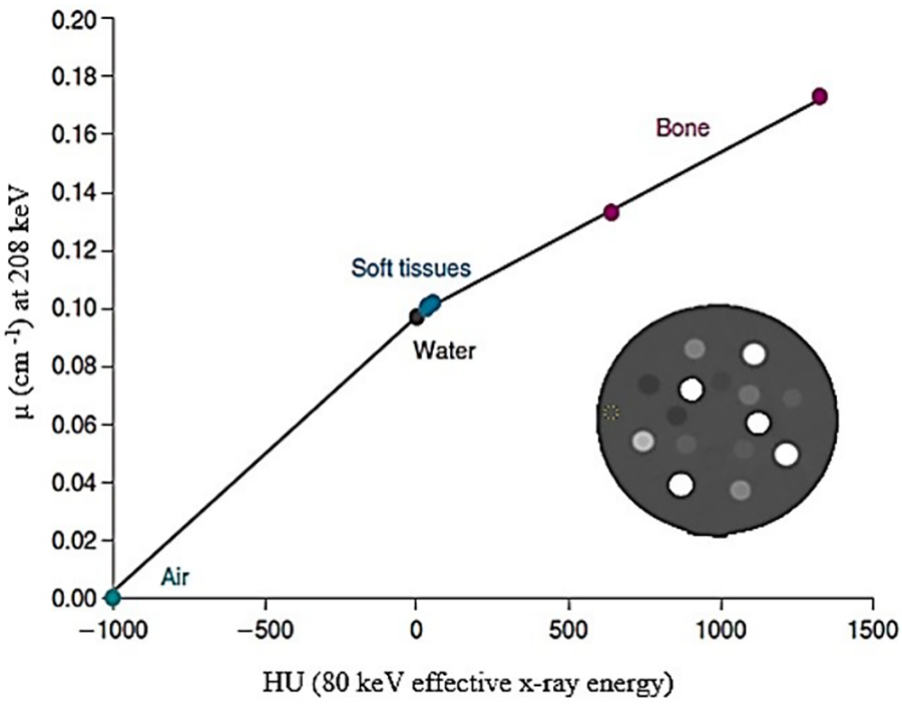
An example of a bi-linear model used to map the effective CT energy to the imaging emission energy of the radionuclide of interest.

Attenuation is the most significant factor that reduces quantitative accuracy, particularly in large patients (165). The routine implementation of attenuation correction has been made easier by the spatially and temporally co-registered PET/CT and SPECT/CT data. Accurate SPECT (or PET) and computed tomography (CT) data registration is necessary to maintain the integrity of the attenuation correction from the CT data (166). Converting the CT images into acceptable attenuation maps using linear attenuation coefficients is the first step in attenuation correction. This entails employing bi-linear models (167), as shown in the example for ^177^Lutetium depicted in Figure 7, to map the effective CT energy to the radionuclide's primary emission energy. Increased CT numbers in patients with metallic implants and those who have taken contrast agents lead to inaccurate SPECT attenuation coefficients, which in turn causes overestimation of radioactive uptake and false-positive results (133).

Advances in AI technology have made it possible to make improvements in attenuation and scatter correction for PET and SPECT scans. AI enhances PET/magnetic resonance imaging (MRI), as well as PET-only and SPECT-only gamma cameras, by facilitating the use of synthetic attenuation maps derived from uncorrected emission images. This eliminates the necessity for a CT scan for attenuation and scatter correction. Accurate attenuation correction maps produced by AI for myocardial perfusion SPECT imaging have been demonstrated (168). Moreover, AI-based attenuation correction prevents misregistration artifacts and simplifies identifying artifacts for physicians (169). Attenuation correction using deep learning-based methods has shown promising results in quantitative SPECT imaging (169–171).

#### 2.3.3 Scatter correction

It is assumed that a photon is eliminated if it is completely absorbed or scattered by the linear attenuation coefficients that are produced using CT data for corrections (172). To increase the quantitative accuracy, scatter correction seeks to eliminate scattered gamma rays that have occurred away from the emission location. Multiple window scatter compensation approaches have practical applications, therefore most gamma camera vendors conduct scatter correction for SPECT data using methods such as the dual-energy window (DEW) (173) and the triple-energy window (TEW) (174). Down-scatter from higher energy gamma rays than those for which the imaging window was set is considered by the TEW, while the DEW only considers the self-scatter from the source. The process of scatter correction involves deducting the predicted scatter projections from the primary photopeak projection image, on a pixel-by-pixel basis. The photopeak and sub-windows record distinct spatial distributions of the scattered gamma rays, and the gamma rays in the sub-windows do not undergo the same scattering as those in the photopeak windows. Negative counts and noise amplification are possible outcomes of the subtraction procedure (175).

In the reconstruction process, scatter may be modeled using the effective source scatter estimation (ESSE) approach (176, 177). To estimate the scatter contribution in the photopeak, the ESSE makes use of pre-calculated scatter kernels of a point source at a specified distance from the collimator face in a uniform water-filled slab phantom based on MC simulations. The 3D OS-EM iterative reconstruction may incorporate the ESSE scatter kernels to account for the scatter. According to reports, the ESSE produces quantification accuracies that are superior to those of the TEW and DEW scatter correction approaches (139). Although practical, multiple energy window methods for scatter correction involve subtracting counts which may lead to reduced image sensitivity. AI has emerged as a promising solution for scatter correction, where the scatter sinogram can be generated from the raw data of emission and attenuation data obtained from PET or SPECT, or it can be created using uncorrected PET images as input data (178–180). AI promises to improve both patient throughput and image reconstruction speed when it is applied to scatter correction (181).

#### 2.3.4 Collimator detector response

**Figure 8:**
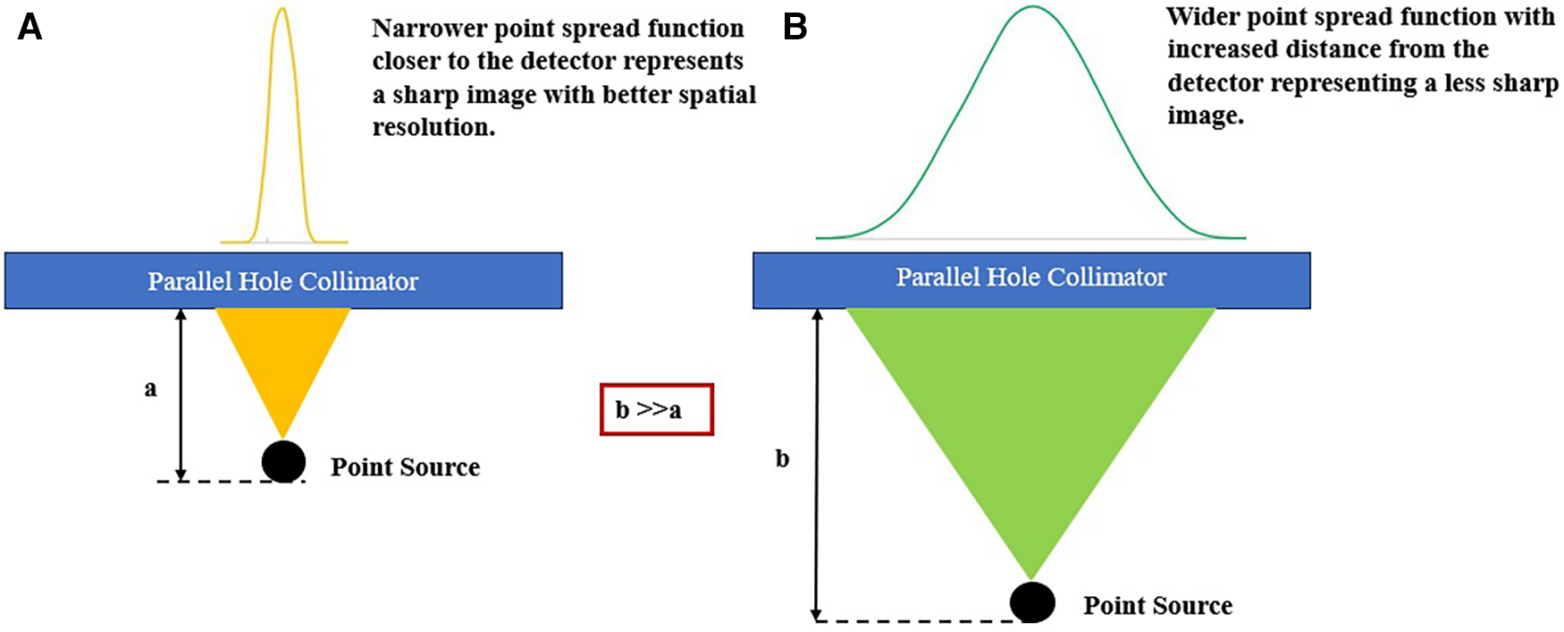
Illustration of the SPECT collimator-detector response determined by the source-detector distance. (**A**) Narrow point spread function closer to the detector. (**B**) A broader point spread function extends further from the detector.

Source-to-detector distance determines the CDR, which is the primary factor affecting the spatial picture resolution in SPECT images, as depicted in Figure 8 (119). Gamma rays pass through and interact with the collimator and detector of the gamma camera, resulting in three factors that alter the CDR (182). The collimator resolution, which is based on the geometrical acceptance angle of the collimator holes and decreases linearly with the source-to-collimator distance, is the first factor (172). The second factor is the detector's intrinsic resolution. To precisely estimate gamma ray interaction positions, this component is restricted by the uncertainty present in the crystal and gamma camera positioning electronics. The probability that the gamma rays will penetrate the septa makes up the third factor.

When gamma rays scatter in the collimator septa and are detected in the main energy window, this is known as septal scatter. Each of these components exacerbates the blurring of the image and reduces the spatial resolution. The effects of medium- and high-energy gamma rays are more noticeable in terms of dispersion and septal penetration. These factors reduce the quantitative accuracy by affecting the resolution of SPECT-reconstructed images of individual pixels and the capacity to identify microscopic diseased tissue, such as metastasis.

The geometric response, which consists of the first two components mentioned above, is easily compensated for by most commercial systems as part of the CDR during the iterative reconstruction process. The manufacturer sets the specifications of the modeling. Increased quantification accuracy has been reported for ^177^Lutetium spheres positioned in a phantom, employing MC modeling scatter compensation techniques similar to the convolution-based forced detection scatter correction which incorporates collimator and detector modeling (126).

CDR correction partially mitigates activity spill-out by reducing the poor resolution effects that cause PVEs. It is important to note that the Gibbs ringing artifacts could appear in the vicinity of sharp boundaries of activity distributions while employing CDR (183). Even while CDR compensation helps minimize PVEs, partial volume corrections (PVCs) still need to be applied to the reconstructed images near the gamma camera resolution limit to improve the accuracy of activity quantification (184).

#### 2.3.5 Partial volume effect

The PVE refers to the phenomenon when activity concentration from an emission reconstructed image is not only confined in the respective voxel but also smeared out into the neighboring voxels. This occurs when an imaged object is similar in size to a multiple of the gamma camera's estimated spatial resolution assessed in terms of its full width at half maximum (FWHM), that is, less than 2 × FWHM (173). Image blurring of objects below the aforementioned resolution volumes is a result of the gamma camera's limited spatial resolution and image sampling in an image matrix, causing the PVE.

**Figure 9:**
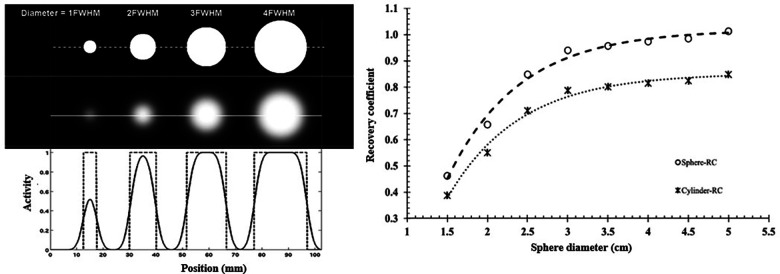
(**A)** Profiles drawn through reconstructed spheres of varying sizes above and below the gamma camera resolution limit, with the spread functions illustrating the activity underestimation. (**B**) Illustration of a recovery coefficient used for partial volume correction (120, 187).

The measured activity distribution is said to be underestimated by the PVE, illustrated in Figure 9A, which is commonly observed in tumor imaging (185). This is one of the challenges in determining dose-response relationships in RPT for tissues other than OAR, which directly impacts dosimetry (186).

The RC is one of the PVC techniques describing the ratio between the actual and measured activity concentration (188). For well-defined shapes, often spheres, an RC curve can be produced as a function of object size (Figure 9B). In phantom investigations where the true activity and object size can be determined, this can be used to readily generate characterization of the gamma camera's PVEs for particular shapes. Nonetheless, this compensatory technique might provide challenges in clinical investigations when the sizes of the organs and tumors are unknown and have irregular shapes. Higher-resolution modalities that include CT or MRI examinations may be employed to determine the size of the tumors and organs. However, since every clinical study would be different, modeling partial volume for all irregular geometries would be cumbersome.

Dosimetry evaluated with a SPECT clinical example ranged from >99% underestimation in the smallest lesion (4 × 5 mm) to more than 60% underestimation in the greatest lesion (28 × 22 mm) (189). Several PVC quantification techniques were examined for ^177^Lutetium, in a 3D-built kidney phantom featuring cortex and medulla compartments (190). The authors recommended against using a sphere-based RC for characterizing organs like the kidney and instead using a geometry-specific RC. Large variations in PVE magnitude are caused by variances in kidney shape. Moreover, the distribution of intrarenal activity has a significant impact on the severity of PVE.

Consequently, it is highly unlikely that RCs created from simpler phantoms will be adequate to rectify the PVE in patient images. Furthermore, renal RCs have been reported to be well-modeled by the surface area-to-volume ratio; this method may also be used for other geometries (191). Numerous software-based PVCs have been proposed (119) but, due to their complexity, they have not been widely used in clinical settings. This explains why sphere-based RCs for PVC are still commonly used, particularly in the measurement of tumors. When assessing radionuclide uptake *in vivo*, the PVE is still a significant component, particularly in small volumes. It will be especially challenging to estimate absorbed doses from RPT accurately until robust methods to account for the PVE are developed.

#### 2.3.6 Volume of interest definitions

To achieve accurate activity quantification the VOI definition is another crucial factor to take into account (192, 193). There isn't a widely recognized technique for defining VOI in NM images. A common approach has been to employ anatomical CT data from SPECT/CT images, a method that has been extensively used in clinical and phantom investigations (128, 137, 152, 190, 194). SPECT quantification accuracy is affected by errors such as misdefinition (variability in organ delineation) and misregistration (between emission and transmission data) errors. Notably, even a single voxel misdefinition can lead to a quantification error of up to 8% (133). These errors become more pronounced in small organs with low levels of activity uptake. While a fully automated segmentation method using CT images and convolutional neural networks has shown accelerated organ segmentation and high accuracy in kidney dosimetry for ^177^Lutetium, RPT, it still necessitates expert supervision and corrections, primarily due to misalignments in the co-registration of SPECT and CT images. Image sampling affects the variability in the VOI definition, and the precision of the quantification accuracy may be improved when using smaller pixels (195). As new radiotracers are consistently introduced into NM dosimetry, the utilization of automated AI-based segmentation is increasingly seen as an advantageous initial step in the dosimetry process (196).

Compensating for the aforementioned factors contributes to an improved estimation of the activity distribution. The selection of appropriate correction techniques depends on various factors, including the specific clinical study under investigation, the accessibility of the correction methods, and the radionuclide under consideration. To assess the accuracy of activity quantification, the percent difference between the actual activity distribution and the quantified activity distribution obtained from the acquired images is estimated. The accuracy of dosimetry is directly linked to the accuracy of the quantified activity.

## 3 Radiopharmaceutical dosimetry

The physical measure used to assess the effects of ionizing radiation in tissue is the absorbed dose $\bar{D}$ (27), which is often used to characterize the energy delivered in a volume with a specific mass. Gray ((J)⁄(kg)) is the international system unit used to express absorbed dose. From this, it follows that the definition of the mean absorbed dose $\bar{D}$ is given by Equation (1).

(1) $$\bar{D}=\frac{d\bar{\varepsilon }}{dm}$$

Where $d\bar{\varepsilon }$ is the mean energy imparted and dm is the mass of a specific tissue volume. Equation (1) is extended to account for the different source-target relationships and the radionuclide decay characteristics expressed in Equation (2).

(2) $$\bar{D}(r_{T},\;r_{S})=\sum_{r_{S}}\tilde{A}(r_{T},r_{S})S(r_{T},r_{S})$$

**Figure 10:**
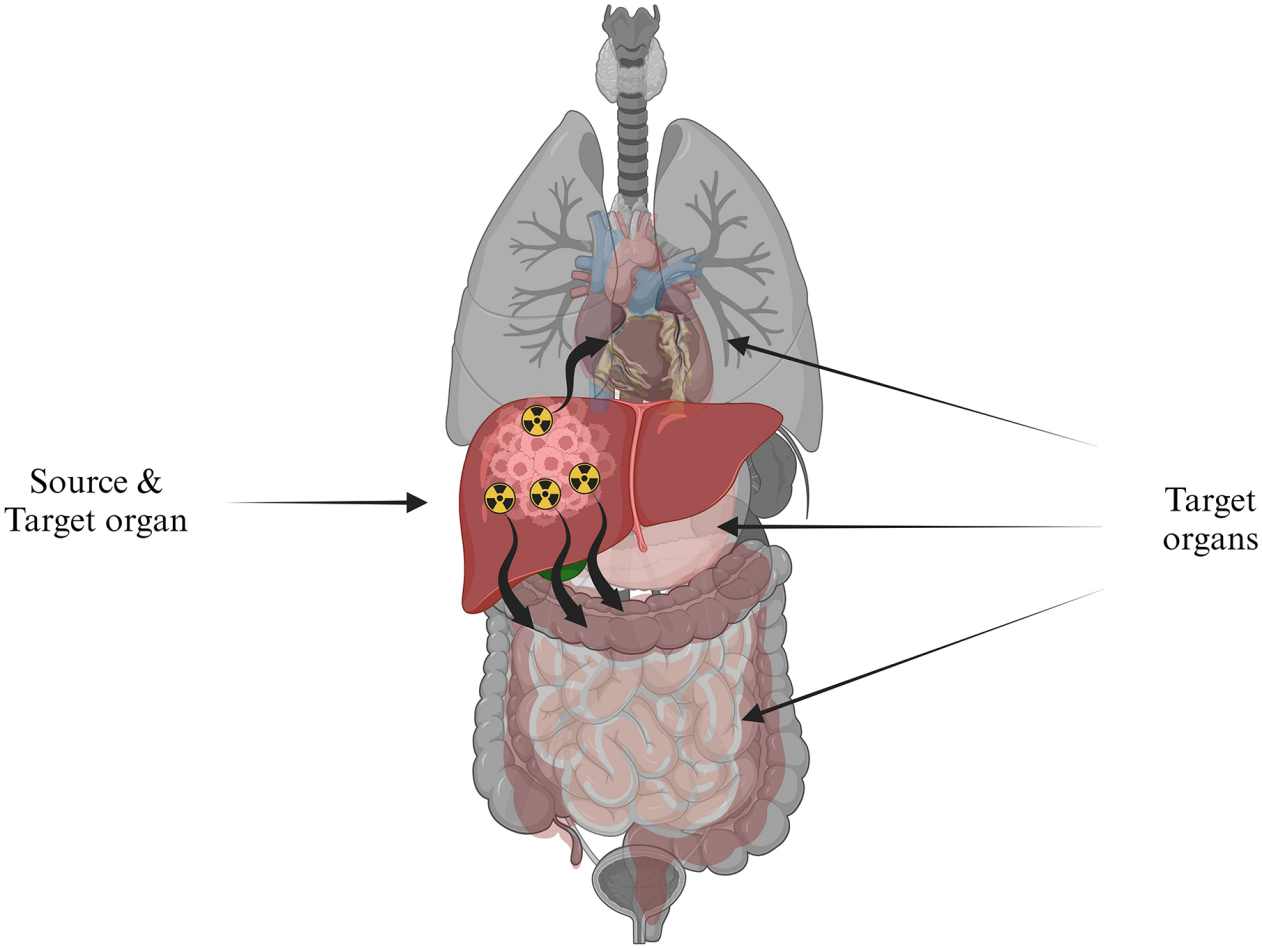
The absorbed radiation dose directed to target organs, symbolized by healthy tissue, originating from a source organ, which is depicted as cancerous cells in the liver containing radioactivity.

Where $\tilde{A}(r_{T},\;r_{S})$ is the time-integrated activity (TIA), which depends on the bio-kinetics of the activity distribution and represents the cumulated activity for a specific period, while $S(r_{T},r_{S})\;$ is the mean absorbed dose deposited in the target organ per TIA unit present in the source organ (Figure 10). Equation (2) forms the foundational basis for RPT dosimetry. This basis was initially developed and presented in the 1960s by the Medical Internal Radiation Dose (MIRD) Committee of the Society of Nuclear Medicine, known as the MIRD formalism. The MIRD formalism gained wide acceptance as the standard method for absorbed dose calculations (197). The absorbed dose computation is the product of two quantities, the imparted energy summations over all emission types (S-coefficient) and the cumulative activity (TIA), which will be elaborated upon in the subsequent discussions.

### 3.1 Time activity data

#### 3.1.1 SPECT/CT images for time activity curves

SPECT/CT images have been widely recognized for their improved capability to estimate activity distributions (124, 129, 198). In the process of activity quantification, the count rate per voxel in the reconstructed SPECT image is proportional to the activity concentration. This proportional relationship is achieved given that previously mentioned corrections are applied (199), encompassing scatter correction using the TEW method (119, 200), CT-based attenuation correction, CDR compensation (119, 201), and corrections for PVEs using RCs (133). The corrected count rate per voxel is then divided by the calibration factor [(counts per second per voxel)/(kilo becquerels per milliliter)] to determine the activity concentration. Even with improved accuracy, the repeated acquisition of multiple SPECT/CT images for dosimetry can be time-consuming for clinical facilities and can impact patient throughput. Consequently, caution should be exercised in the selection of optimal image sampling schedules, as suboptimal choices may lead to significant over- or underestimations of absorbed dose estimates, particularly for organs at risk such as the kidneys (62). It is crucial to understand the purpose of dosimetry and the resources available in a clinical setting to achieve accurate results. While simplifications in dosimetry methodology may affect its accuracy, an acceptable level of uncertainty can be determined based on clinical needs. Personalized dosimetry often requires SPECT/CT studies at multiple time points, but efforts have been made to reduce the burden on patients and clinics. In centers with limited capacity, dosimetry can be performed at alternate cycles or by using less quantified images for subsequent cycles. The use of only the initial cycle, as well as post-therapy imaging, can be developed into a quantitative image for absorbed dose estimations. Gamma camera availability limitations can be addressed by using a hybrid approach of WB/SPECT. Pre-therapy images can predict therapeutic absorbed doses in theranostic applications, allowing for tailored activity prescriptions for optimized therapy success. Hybrid WB/SPECT imaging methods have been introduced to streamline the imaging process for dosimetry (153). Furthermore, the use of single time-point post-treatment imaging for dosimetry, based on SPECT/CT data, has recently been described in the literature; however, its widespread adoption has yet to be achieved (202).

### 3.2 Time activity curve

The TAC gives temporal information regarding the patient-specific variations in radiopharmaceutical uptake, retention, and excretion. Successive quantitative images obtained at various time points after the administration of radiopharmaceuticals are used to generate TACs. If the TAC is not determined optimally, it could lead to considerable dosimetry inaccuracies. Once the TACs for various source organs of interest are obtained, the TIA is determined by integrating these curves. The accuracy of the TAC is influenced by the number of imaging time points, the frequency of the image sampling schedule, the appropriate imaging span period following administration (integration period), and the model applied for TAC fitting, especially for TIA calculations (203–205). For accurate results, at least three data points should be gathered throughout two to three effective half-lives, with the integration time matching the study's biological endpoint (27). Gleisner et al. (206) observed detectable levels of [^177^Lu]Lutetium-DOTATATE five to seven weeks after injection due to tumor retention. Tumor dosimetry at a time point beyond the conventional seven days following administration might be useful.

#### 3.2.1 Time-integrated activity

The TIA, also referred to as cumulated activity, is computed by taking the area under the TAC derived from the series of imaging time points. Multi-exponential functions that are integrated analytically can be fitted to the TACs. Uncertainties in the TAC fitting models have been reported (207). The constants determined from the exponential functions describe the bio-kinetic data in the organ of interest. Statistical analyses have been conducted to determine the best-fitting functions for the TIA (203). The total number of decays (S-coefficient) obtained within a specific volume must be obtained to calculate the absorbed dose once the following conditions have been met: (i) The quantified activity has been accurately determined, (ii) the optimal image sampling has been obtained, and (iii) the optimum fitting model to compute the TIA has been established. Performing two-time point imaging can yield TIA estimates with an average error below 5% of the reference TIA for both tumors and kidneys. Similarly, three-time-point imaging offers a comparable level of error but exhibits less variability (208).

Optimal sampling of hybrid WB/SPECT images for kidney dosimetry has revealed that the variability in TIA depends on the number of post-administration activity images, especially in scenarios with two-time points (166). In such cases, prior knowledge of population averages for biokinetic data is necessary, and this approach may not be suitable if patients' biokinetics deviate significantly from the population average, particularly in the context of toxicity detection. The application of a single time point may deviate from the principle of patient-specific dosimetry for treatment planning.

### 3.3 Absorbed dose computations

**Figure 11:**
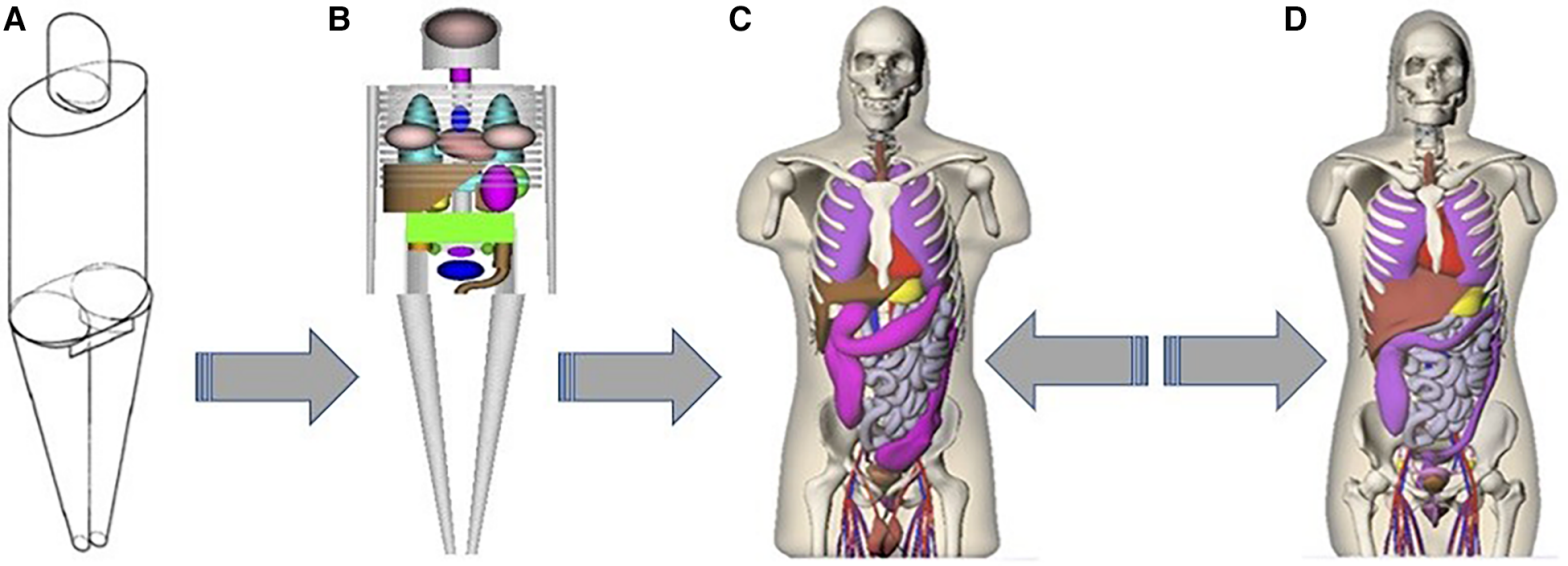
Original stylized adult male model of (212): (**A**) exterior view, (**B**) skeleton and internal organs. Anterior views of the Segars NURBS models (216) (**C**) adult male and (**D**) adult female.

The S-coefficient accounts for the mean energy emitted per decay of the radionuclide, the mass of the target organ, and the absorbed fraction of the energy emitted from the source organ. The absorbed fraction depends on the type and energy of the emitted radiation, the size, shape, and composition of the source and target regions, and the distance and type of material separating the source-target regions. The S-coefficients are stored as look-up tables for a variety of radionuclides and multiple source-target organ combinations (209). The MIRD schema is commonly used for organ-level S-coefficient estimates (210). The dosimetry models employed for the computation of S-coefficients have advanced from simple geometric shapes to more intricate voxel-based phantom series using NURBS models, as illustrated in Figure 11 (211, 212). These models rely on standardized published organ masses (213, 214). More accurate patient-specific dosimetry can be achieved if the S-coefficients are scaled by the mass of the patient's organ. The progression of these models, encompassing various source and target organs, has been well-documented (215).

**Figure 12:**
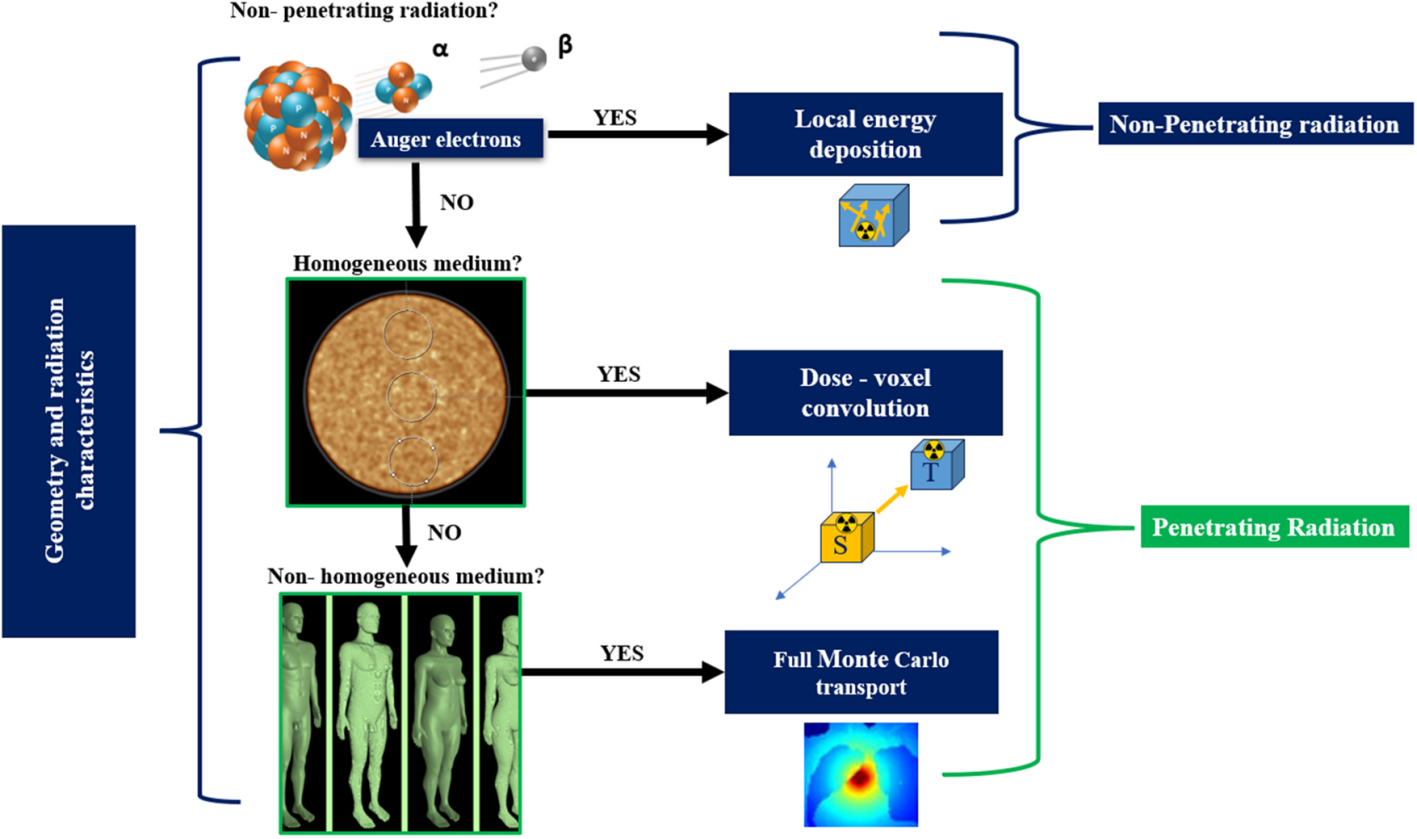
A selection criteria for choosing an appropriate dosimetry algorithm (218, 219).

Three approaches are available to compute the S-coefficients, namely, local energy deposition (LED), convolution using dose point kernels (DPK), and direct (full) Monte Carlo transport. A selection criterion, detailed in reference (102) and illustrated in Figure 12, may serve as a practical guideline for selecting the most suitable algorithm for absorbed dose calculations. To elaborate on the guidelines, if the radiation in question is non-penetrating, LED may be an appropriate choice. However, for penetrating radiation with a high emission yield, where energy deposition from radiation sources outside the original volume could contribute significantly to cross-doses, the other two algorithms should be considered. In instances where radiation propagation within tissue is homogeneous, the behavior of LED is primarily influenced by the distance from the emission point (217). When the propagation medium is homogeneous, but radiation particles penetrate the tissue, convolution with DPK can be a viable approach. When dealing with penetrating radiation in a non-homogeneous medium, direct MC methods should be considered, particularly for accurate absorbed dose estimates.

#### 3.3.1 Local energy deposition

A simple method to calculate absorbed dose is the LED which only considers self-dose within the voxel and assumes that all produced energy is entirely deposited in the originating voxel. Absorbent fractions are set to one using this method (220). The range of the charged particles released during radioactive decay, i.e., the alpha, beta particles, and Auger electrons, which are the primary contributors to energy deposition, determines the validity of this assumption (221). The assumption is valid for the majority of radionuclides used in RPT, where the projected path length of charged particles in tissue is less than voxel dimensions (128). It is also applicable for ^177^Lutetium, given that the charged particles' range is typically within the dimensions used for clinical SPECT images (153). Even for radionuclides such as ^90^Yttrium, where the range of emitted charged particles is longer, most of the emitted charged particles remain within a projected path length of 5 mm, which aligns with SPECT voxel dimensions.

The validity of the LED assumption is contingent upon the gamma-ray yield, making it less applicable to gamma rays. Because the ^177^Lutetium 208.4 keV gamma ray has a relatively low yield (10.4%), there is a low possibility of cross-dose contribution from target organ gamma rays. This makes the method particularly accurate for assessing ^177^Lutetium toxicity studies (222–224). Radionuclides such as ^131^Iodine with higher gamma-ray yield a higher probability of cross-dose from the target organ. As a result, different approaches such as convolution with DPK (225, 226) and direct MC transport calculations (128, 227–230) are considered.

#### 3.3.2 Convolution with point voxel kernels

Convolution with DPK is considered when photons have a path length that is longer than the spatial resolution of the reconstructed SPECT image. This approach employs a DPK that describes the radial absorbed dose in a uniform water medium when an isotropic point source is positioned at the center. The total energy released per unit mass (TERMA) in conjunction with the kernel is used to calculate the dose (19, 231). The TIA image is convolved with these functions to estimate the deposited energy distribution, thereby deriving the absorbed dose (232). Instead of calculating a continuous dose point kernel, a discrete DPK is often determined. The TIA image is then convolved with tissue-specific DPKs (233).

Originally, these methods used simulations in homogeneous phantoms (234, 235) that included comprehensive decay data for photons and mono-energetic charged particles. The techniques have since been expanded to density scaling for non-homogeneous tissue ^177^Lutetium (225, 226, 236). The methods proved less accurate when applied to a non-homogeneous medium. For inhomogeneities, DPKs have been implemented and compared with the direct MC gold standard (233). Due to the inadequate kernel size used in DPK dosimetry, the modified DPK overestimated the mean absorbed dose from the MC technique by 5% to 8%. Neural networks have been trained with DPKs to estimate absorbed dose calculations in kidneys (189, 237).

An AI-based deep learning approach for whole-body organ level dosimetry considering tissue inhomogeneity and patient-specific anatomy has also been described (238). In this novel method using patient-specific anatomy and the S-coefficient kernel, deep learning was used to predict the energy deposition and compared well with the direct MC dosimetry. Additionally, the techniques were marketed as a way around MC dosimetry's computational burden restriction. Even though deep learning-assisted dosimetry is a very promising advancement in absorbed dose estimation, the outcomes rely on how many training kernels must be assembled for a sizable patient cohort, all relevant tissue combinations, and a vast number of voxels to minimize inter-patient variability. The methods rely on simplified computations to estimate absorbed doses, as accounting for the complex effects of tissue heterogeneity in human anatomy on energy propagation and deposition proves challenging.

#### 3.3.3 Direct Monte Carlo-based dosimetry

The complete meaning of patient-specific dosimetry entails a detailed evaluation of the absorbed dose in tumor and normal tissue provided by the MC-based pre-calculated S-coefficients that are tailored to the unique anatomy and heterogeneity of each patient. Direct MC-based absorbed dose calculations are accepted as the gold standard (239). However these methods are still computationally demanding for widespread clinical application and, as a result, have received limited acceptance as a standard for clinical dosimetry (128, 134). However, the concept has potential due to the rapidly increasing computational capacity.

In addition to computing patient-specific S-coefficients, MC absorbed dose calculations are capable of handling non-uniform absorbed dose estimates at a voxel level. This allows them to overcome the assumptions involved in the previously described approaches. Particle transport is simulated by the CT images, while radiation decay characteristics are simulated by the SPECT images. MC-based dosimetry uses predefined patient atomic composition and density information from CT images to model the emission from SPECT images and the transport of photons as well as charged particles across different structures in the body. To sample decay locations, it is assumed from the quantified SPECT images that each voxel represents activity from the relevant volume element in the patient. This is one of the reasons that accurate activity quantification is important. The density image volumes simulate the passage of charged particles and photons. In an image matrix with the same voxel dimensions as the reconstructed SPECT image, the energy deposition at each interaction site is scored to create the absorbed dose rate images (128). The patient's unique geometry can be used to construct source-target combinations rather than relying on reference phantom models. The S-coefficients are computed for each radiation source to the target combination and consider the radiation range for each source-to-target geometry (217). In addition to accounting for tissue heterogeneity and secondary particle emissions, MC dosimetry also takes tumor geometries and tissue type transitions into consideration (226, 240).

Small-scale dosimetry, which involves calculating radiation absorbed dose at sub-organ and sub-tumor levels, may be addressed using DPK and direct MC radiation transport simulations. In addition to accounting for tissue non-homogeneities, another advantage of MC simulation is its applicability to conditions where charged-particle equilibrium is not achieved, such as tissue interfaces (241). To use DPK in heterogeneous media, simple scaling factors are applied to those in water-equivalent media, producing results that closely approximate those of MC but with reduced computational times. Voxel dosimetry involves calculating radiation absorbed dose to tissue regions ranging from a few centimeters to hundreds of micrometers. This method is commonly associated with tomographic imaging such as PET/CT and SPECT/CT or autoradiographic techniques for activity quantification. However, in the context of multicellular, cellular, and subcellular dosimetry, there is a need for quantifying activity at smaller scales, typically ranging from tens to hundreds of micrometers (242). Acquiring activity distribution data directly from clinical tomographic gamma camera images at these small scales remains challenging due to the gamma camera's limited spatial resolution. Nonetheless, autoradiographic techniques may offer a solution by enabling the quantification of activity within groups of cells (multicellular) and even within single cells. In preclinical settings, small-scale or voxel dosimetry has become a more widely utilized approach due to the improved resolution capabilities of preclinical imaging (112). However, it's worth noting that conventional preclinical dose estimations often assume uniform distribution of activity and dose deposition within organs. This assumption may not reflect reality, particularly for *β*-/*α*-emitting radiopharmaceuticals, where tissue activity distribution can be heterogeneous.

Tissue non-homogeneities may be represented using BEDs and dose-volume histograms (DVHs) unique to each patient. DVHs demonstrate the relationship between a volume (%) that has received a specific absorbed dose as a function of the absorbed dose (27). In EBRT, DVHs are frequently employed to depict the dose distributions of tumors and OAR. It is unclear how to appropriately present the idea of DVHs as a reporting mechanism in the NM community (243). Applications of DVH and BEDs are still being developed for NM due to the limited spatial resolution of SPECT/CT images that do not support fine sub-region uptake (244). The use of DVH and BED in the NM clinics has been restricted due to the limited extent of their clinical validation.

Ideally, a dosimetry program should encompass all the necessary steps required in the clinical dosimetry workflow, commencing with dose calibrator and gamma camera calibrations and concluding with computation of dose estimates. This facilitates a modular approach, and the workflow becomes more user-friendly with incentives to complete all steps in a clinical context required to obtain accurate dosimetry. Several commercial software programs, listed in Table 3, are available for RPT dosimetry. These tools are based on different scientific methodologies comparable to the ones discussed above and have become valuable tools for clinical applications involving ^177^Lutetium dosimetry. It's worth noting that not all commercial dosimetry program encompasses every step of the dosimetry workflow. To enhance the precision of dose estimations, it is advisable to employ optimization techniques at each stage of the dosimetry process to minimize errors (207).

**Table 3:** Commercially available dosimetry software programs.

Dosimetry software	Dosimetry method	References
OLINDA/EXM™ 2.0	RADAR (voxel-based realistic human computational phantoms)	(245–247)
PLANET® Dose (DOSIsoft™)	Local energy deposition	(210, 248)
GE™ Healthcare Dosimetry Toolkit	OLINDA/EXM™	(210, 249)
MIM Sure Plan MRT™	Convolution	(210, 250)
Torch™	Monte Carlo method	(210)
Voxel Dosimetry™	Monte Carlo method	(251)
QDOSE®	IDAC-Dose 2.1 Convolution	(252)

*RADAR*, Radiation dose assessment resource; *OLINDA/EXM*, Organ level Internal dose assessment/exponential modeling.

## 4 Practical prospects

RPT is an evolving field that requires continuous refinement. Its clinical significance involves comparing the administered activity tailored to individual patients with generic or fixed-activity regimens, demonstrating its impact on clinical outcomes. This strategy will help collect the crucial data needed for establishing the dose-response relationship in RPT. The focus of these initiatives ought to be on well-planned, multi-center clinical trials that compare the one-size-fits-all approach with dosimetry-based activity regimens. Following the defined tailored treatment in EBRT, patient-specific dosimetry-driven activity administrations ought to be given priority as standard procedures. Technological developments in imaging hardware resolve some of the historical issues that have been barriers to this endeavor. PET gamma cameras with full-ring detector geometries have made whole-body imaging more rapid and practical, while SPECT gamma cameras with solid-state detectors offer better energy resolution. Harmonizing the crucial first steps in the dosimetry chain holds the key to generating consistent dose estimates using patient-specific dosimetry. The calibration of dose calibrators and precise determination of gamma camera calibration parameters should be the first steps in the pursuit of this objective. These are easy but necessary first steps in obtaining reliable and consistent dosimetry readings.

## 5 Conclusion

There is growing evidence demonstrating inter-patient variability, suggesting that minimizing OAR toxicity and optimizing tumor management must be balanced. Despite these progressions, a fixed administered activity regime continues to be predominant, primarily because of uncertainties associated with dosimetry calculations for RPT. This state stems from several obstacles including the lack of standardized methods in the clinical dosimetry workflow, the arduous effort for successive imaging examinations, and the lack of comprehensive documentation correlating the administered activity to patient outcomes.

While patient-specific dosimetry plays a significant role in ensuring safety, its application lacks empirical support, a notion reinforced by the “tolerated holy gray” of maximum threshold values. Consequently, the more established empirical activity administration is often preferred over patient-specific dosimetry-driven activity administrations, which are perceived as complicated and time-consuming (253). Patient-specific dosimetry provides the evidence-based data necessary for personalized RPT, making it essential to achieving RPT's full potential as a precision-based cancer therapeutic alternative. The pursuit for personalized administered activities in RPT must remain a priority, guided by Einstein's dictum to be “…as simple as possible but no simpler.”
